# Cutaneous sarcoidosis simulating porokeratosis of Mibelli

**DOI:** 10.11604/pamj.2015.20.195.5003

**Published:** 2015-03-03

**Authors:** Fatima Zahra Elfatoiki, Wessal Soussi, Soumia Chiheb, Lamia Jabri, Hakima Benchikhi

**Affiliations:** 1 CHU Ibn Rochd, Service de Dermatologie, Casablanca, Maroc; 2 Centre de Pathologie Casapath, Casablanca, Maroc

**Keywords:** Sarcoidosis, porokeratosis of Mibelli, granuloma

## Abstract

We report a skin localization of systemic sarcoidosis, which presented with lesions that resemble porokeratosis of Mibelli. Skin biopsy showed non-caseating sarcoidal granuloma. Whereas cutaneous sarcoidosis is present in up to one-third of cases and may present with a wide variety of lesions, our presentation is uncommon. Partial remission was obtained with hydroxychloroquine and prednisone

## Introduction

Sarcoidosis is a systemic disease that can involve almost any organ system. Cutaneous manifestations are very heterogeneous and infiltration with non caseating granulomas is the hallmark of the disease. We report a case of cutaneous sarcoidosis mimicking porokeratosis of Mibelli.

## Patient and observation

**Figure 1:**
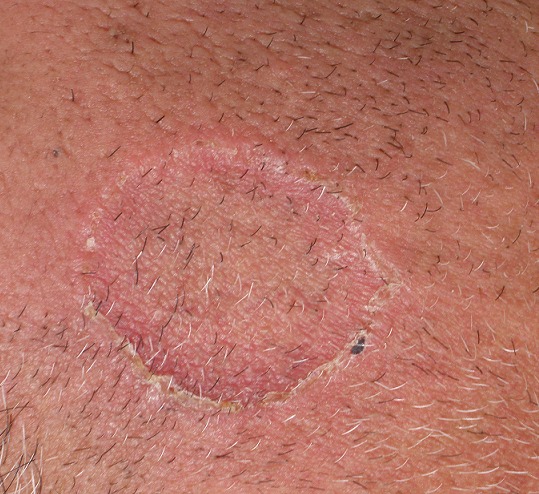
Erythematous annular lesion of the left cheek, with well-demarcated keratotic and filiform border

A 52-year-old man (skin type 4) with no excessive sun exposure, with type 2 diabetes mellitus treated with metformin for 4 years, presented for 3 years two symmetrical lesions of the face. Skin examination showed erythematous annular lesion of each cheek, measuring 3 cm in diameter, with well-demarcated keratotic and filiform border (Figure 1). The rest of the tegument was normal and physical examination was otherwise normal. Histological examination of a cutaneous biopsy showed dermal non-caseating sarcoidal granulomas with few lymphoid cells around epithelioid cells (Figure 1). The Ziehl-Neelsen and PAS staining techniques were negative. No cornoid lamella was noted. Serum Angiotensin Converting Enzyme rate was slight increased and tuberculin skin test (PPD test) was negative. Liver function, kidney function, hemogram, serum and urinary calcium, electrocardiogram, hands and feet X-rays without abnormalities. Chest radiograph showed a fine reticular opacity of the lower third of lung parenchyma. Chest computed tomography demonstrated bilateral hilar and pretracheal adenopathies associated with fine reticular bands in the lower lobes basal segments. Spyrometry revealed light restrictive disorder and the ophthalmologic examination was normal. Treatment with prednisone 0,5mg/kg/day associated with hydroxychloroquine 400mg/day was initiated with partial remission after 3 mounths.

**Figure 2:**
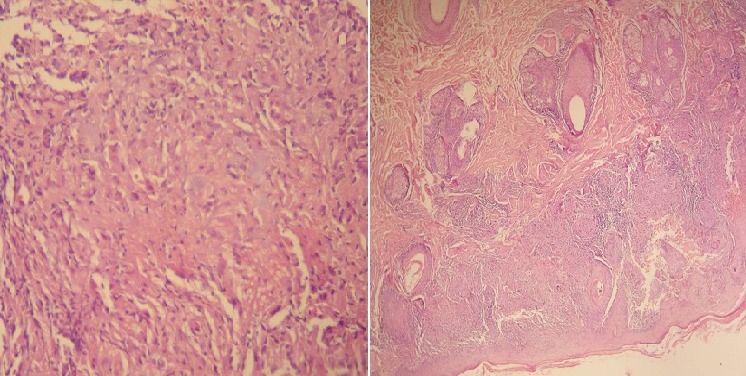
Dermal non-caseating sarcoidal granulomas

## Discussion

Sarcoidosis is a multisystem granulomatous disease that most often affects the lungs and intrathoracic lymph nodes. Cutaneous involvement occurs in 20-35% of cases and usually may be the only organ affected [1]. Skin lesions are divided into non-specific inflammatory types which the most common is erythema nodosum, and specific lesions which skin biopsy showed non-caseating sarcoidal granuloma formed by concentrical disposition of epithelioid cells [2]. Specific sarcoidal skin manifestations are very heterogeneous and cutaneous sarcoidosis is another great imitator [1]. Lesions may occur as macules, papules, plaques, nodules, ulcers, infiltrated scars, subcutaneous lesions infiltrated erythroderma and scarring alopecia. They may be skin-colored, yellow-translucent, hyper-or hypopigmented or erythematous-to-violaceous in color. In addition, epidermal changes may be absent or include atrophy, hyperkeratosis, or telangiectasia [2]. In our case the diagnosis of cutaneous sarcoidosis is established by clinical, histopathologic features, the presence of pulmonary involvement and increased serum rate of Angiotensin Converting Enzyme. Sarcoidal cutaneous presentation mimicking porokeratosis of Mibelli has never been reported. The lesions were erythematous and annular with well-demarcated keratotic and filiform border located on the cheeks. Histological examination of a cutaneous biopsy showed the presence of sarcoidal granuloma without any cornoid lamella confirming the diagnosis of sarcoidosis. Our case confirms the histomorphological variation of lesions in cutaneous sarcoidosis. We believe that this presentation of cutaneous sarcoidosis underscores the importance of comparing the clinical and the pathological features for appropriate patient care. Careful clinical and pathological correlation led to the proper diagnosis and therapy.

## Conclusion

The dermatologist plays a critical role in elucidating the clinical diagnosis and assisting other specialists in the investigation of a systemic disease.

## References

[1] Tchernev G, Patterson JW, Nenoff P, Horn LC (2010). Sarcoidosis of the skin: a dermatological puzzle: important differential diagnostic aspects and guidelines for clinical and histopathological recognition. J Eur Acad Dermatol Venereol..

[2] Marchell RM, Judson MA (2010). Cutaneous sarcoidosis. Semin Respir Crit Care Med..

